# Feasibility and usability of a regional hub model for colorectal cancer services during the COVID-19 pandemic

**DOI:** 10.1007/s13304-022-01264-y

**Published:** 2022-03-03

**Authors:** Filipe Carvalho, Ailín C. Rogers, Tou-Pin Chang, Yinshan Chee, Dhivya Subramaniam, Gianluca Pellino, Katy Hardy, Christos Kontovounisios, Paris Tekkis, Shahnawaz Rasheed, London Colorectal Cancer Hub Network Collaborators, A Karim, A Chung, A Ramwell, R Hagger, N West, L Toquero, A Gupta, P Toomey, A Raja, N Pawa, S Mills, O Warren, C Nicolay, B Thava, N Daulatzai, I Jenkins, D Miskovic, E Burns, A Antoniou, T Agarwal, N Sinha, A Slesser, A Prabhudesai, Y A MohsenMyers, S Harris, S Mohamed, M Abulafi, A Shanmuganandan, J Dellen

**Affiliations:** 1 Department of Colorectal Surgery, The Royal Marsden Hospital NHS Foundation Trust, London, SW3 6JJ UK; 2 Department of Advanced Medical and Surgical Sciences, Università degli Studi della Campania “Luigi Vanvitelli”, Naples, Italy; 3 Colorectal Surgery, Vall d’Hebron University Hospital, Barcelona, Spain; 4 Chelsea and Westminster Hospitals NHS Foundation Trust, London, SW10 9NH UK; 5 Department of Surgery and Cancer, Imperial College London, London, SW10 9NH UK

**Keywords:** COVID-19, Colorectal surgery, Cancer, SARS-CoV-2

## Abstract

The outbreak of the COVID-19 pandemic produced unprecedented challenges, at a global level, in the provision of cancer care. With the ongoing need in the delivery of life-saving cancer treatment, the surgical management of patients with colorectal cancer required prompt significant transformation. The aim of this retrospective study is to report the outcome of a bespoke regional Cancer Hub model in the delivery of elective and essential colorectal cancer surgery, at the height of the first wave of the COVID-19 pandemic. 168 patients underwent colorectal cancer surgery from April 1st to June 30th of 2020. Approximately 75% of patients operated upon underwent colonic resection, of which 47% were left-sided, 34% right-sided and 12% beyond total mesorectal excision surgeries. Around 79% of all resectional surgeries were performed via laparotomy, and the remainder 21%, robotically or laparoscopically. Thirty-day complication rate, for Clavien–Dindo IIIA and above, was 4.2%, and 30-day mortality rate was 0.6%. Re-admission rate, within 30 days post-discharge, was 1.8%, however, no patient developed COVID-19 specific complications post-operatively and up to 28 days post-discharge. The established Cancer Hub offered elective surgical care for patients with colorectal cancer in a centralised, timely and efficient manner, with acceptable post-operative outcomes and no increased risk of contracting COVID-19 during their inpatient stay. We offer a practical model of care that can be used when elective surgery “hubs” for streamlined delivery of elective care needs to be established in an expeditious fashion, either due to the COVID-19 pandemic or any other future pandemics.

## Introduction

SARS-CoV-2 (responsible for COVID-19) was declared a pandemic by the World Health Organization (WHO) in early 2020, and a 3-month nationwide lockdown was implemented in the United Kingdom (UK) on March 23rd in an attempt to reduce virus transmission [1, 2]. With the initial surge of COVID-19 cases, healthcare staff were redeployed outside of usual roles to care exclusively for infected patients, while other staff members were affected or forced to self-isolate due to disease exposure. Hospitals were faced with the downstream challenges of increased numbers of acutely unwell patients in combination with limited bed capacity and critical staff and resource shortages [3, 4]. In recognition of the broader effects of COVID-19 on healthcare delivery, the UK National Health Service (NHS) sought to rationalise resources to create bed capacity and facilitate care for those with COVID-19, focussing on limiting spread of the disease and minimising fatalities. As part of this strategy, elective surgical procedures were suspended on April 15th for three months nationwide [5, 6].

The deferral of elective surgery was not the only factor impacting cancer care in the pandemic. Intensive care units (ICU) were filled with COVID-19 patients, with a resultant lack of post-operative ICU beds for patients requiring emergency surgery [7]. Staff shortages and social distancing measures led to a dramatic reduction in patient access to in-person outpatient appointments and diagnostics. Fear of contracting nosocomial COVID-19 infection led to underreporting of symptoms by patients and hesitancy to present for medical treatment when indicated [7]. COVID-19 exposure in the peri-operative setting is associated with poor post-operative outcomes and rendered surgeons and patients alike reticent to undertake major surgery in the setting of the pandemic [8]. The cumulative effect of these factors contributed to significant disruptions in cancer care and services nationally [9].

The pandemic represented a rapidly evolving scenario, with unprecedented challenges to the healthcare systems globally, and several countermeasures were suggested to provide care to patients with colorectal conditions [9–11]. To ensure continuity of colorectal cancer services during the COVID-19 pandemic, the Royal Marsden Partners Cancer Hub (RM Partners Cancer Hub) was established. This Cancer Hub was collaboratively designed with the sole aim of centralising resources for patients with colorectal and anal cancer across the London region, ensuring optimal delivery of cancer care whilst minimising patient exposure to COVID-19.

The aim of this study is to investigate the feasibility and usability of this bespoke Cancer Hub model in delivering elective colorectal and anal cancer surgery services as a regional collaborative network at the height of the first wave of the COVID-19 pandemic, whilst detailing the steps that led to its inception.

## Materials and methods

This is a retrospective study aimed to describe the strategies adopted at several UK centres to ensure continued care to colorectal cancer patients during the pandemic, and to report on the outcome of the network. The study was conducted following the Strengthening the Reporting of Observational studies in Epidemiology (STROBE) Statement [12].

### Royal Marsden partners cancer hub creation

A collaborative network was devised to provide a centralised surgical pathway for patients with colorectal or anal cancer across the London region during the COVID-19 pandemic. This initiative was created for patients within the catchment area who required time-critical cancer surgery but for whom access to treatment in their local trust was affected by the COVID-19 surge.

Colorectal surgeons specialising in cancer treatment from all NHS trusts in the greater north to south west London area were invited to join at inception. Eligible patients were discussed centrally at a weekly multidisciplinary team meeting (MDT) and categorised by consensus agreement of a quorum of surgeons, with surgical treatment performed at either of two designated ‘COVID-19 free’ surgical sites in London, facilitating other hospitals in the network to focus on pandemic care.

**Fig. 1:**
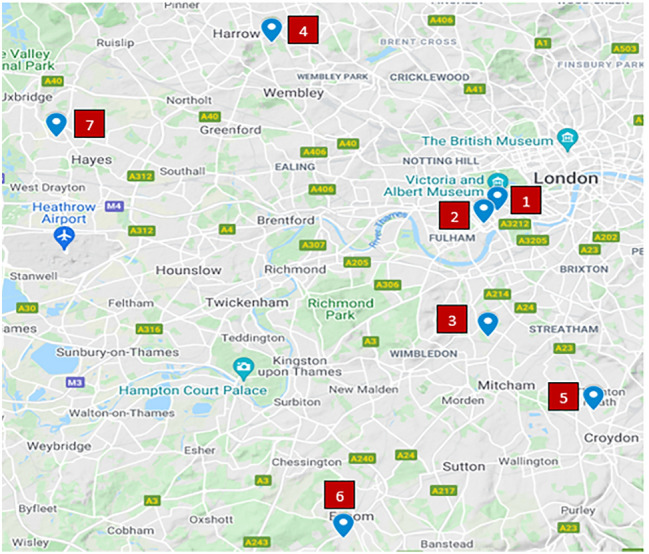
RM Partners Cancer Hub participating London NHS Trusts and geographical location. 1—Royal Marsden Hospital/RM Partners Cancer Hub; 2—Chelsea and Westminster NHS Foundation Trust; 3—St George’s University Hospital NHS Foundation Trust; 4—London North West University Healthcare NHS Trust/St Mark’s Hospital; 5—Croydon Health Services NHS Trust; 6—Epsom and St Helier University Hospital NHS Trust; 7—The Hillingdon Hospital NHS Foundation Trust

The Royal Marsden Hospital (RMH) and BUPA Cromwell Hospital (BCH) were chosen as the ‘COVID-19 free’ sites for delivery of surgical care for several reasons. Both hospitals are located in central/west London, facilitating ease of access for patients and surgeons alike. All patients admitted to or attending either site could be forecasted in advance, allowing self-isolation, screening and testing for COVID-19 prior to any attendance. All of these factors collaboratively ensured that both hospitals could function independently from other NHS units in the area to deliver elective cancer care in a way that was safe for patients, while ensuring ongoing care to acutely unwell COVID-19 patients elsewhere, and thus the RM Partners Cancer Hub was created (Fig. 1).

### Patient selection

**Fig. 2:**
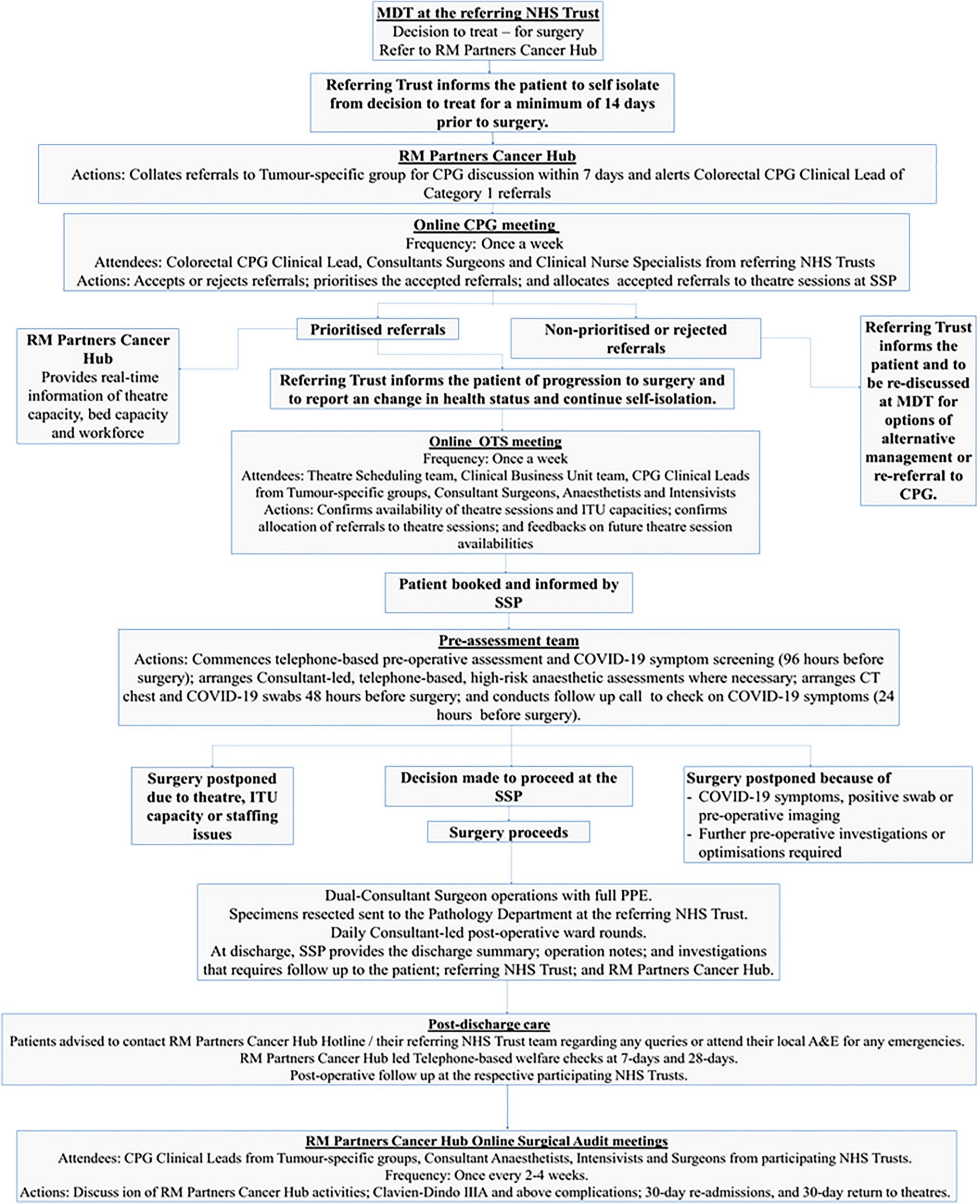
Cancer hub pathway

Patients were included if they had colorectal or anal cancer and were deemed suitable for surgery following completion of diagnostics and MDT discussion at their respective NHS Trusts. Patients were excluded for referral if they had active COVID-19 infection. Referral to the RM Partners Cancer Hub was done using a standardised online referral form. The referring NHS Trusts were responsible for communicating to the patient their decision to offer surgery through the RM Partners Cancer Hub and advising the patients and their respective households to commence self-isolation immediately (Fig. 2).

### Patient prioritisation

Strategic prioritisation of cases for diagnosis and management was employed to mitigate complications attributable to delays. All RM Partners Cancer Hub referrals were listed for discussion within seven days from the date of referral at a centralised Clinical Prioritisation Group (CPG) online meeting. Weekly CPG meetings were chaired by a CPG lead and virtually attended by consultant surgeons or senior clinical representatives from each participating NHS Trust.

Clinical prioritisation was made in accordance with NHS England published guidelines for the management of cancer patients during the COVID-19 pandemic [13], with patients categorised into three priority levels:

- Priority level 1a and 1b patients were those requiring life-saving surgery within 24 or 72 h, respectively. These included patients with clinical presentation of obstruction, perforation or haemorrhage from colorectal or anal cancer.
- Priority level 2 patients were defined as those who needed elective surgery with the expectation of cure within 4 weeks. These included colorectal or anal cancer patients who were at risk of imminent obstruction that were not suitable for stenting; stage 1 to 3 colorectal cancer patients who did not meet requirements for neoadjuvant treatment; and those colorectal or anal cancer patients’ post-chemotherapy and/or radiotherapy who required curative surgery.
- Priority level 3 patients were defined as those who required elective surgery that could be performed within 10 to 12 weeks with no predicted negative oncological outcome.

### Theatre allocation

CPG-prioritised cases were allocated theatre slots in a transparent, mutual and cohesive manner, with agreement between NHS Trust representatives and a CPG meeting quorum of at least four consultant surgeons. The forum was in the form of a virtual video conference meeting to facilitate real-time screen-sharing of theatre calendars for both surgical sites. Cases were consecutively scheduled into available theatre slots on the basis of patient priority and availability of the primary surgeons.

High risk anaesthetic cases or complex surgeries that required the input of multi-visceral surgical teams were performed in RMH, the designated high-risk surgical site provider. The referring NHS Trusts were responsible for communicating the outcome of the CPG meeting to the patient including the date of surgery or its rejection, and for advising the patient and their household to continue self-isolation for at least 7 days prior to their surgery (Fig. 2).

### Pre-operative assessment

The pre-operative assessments of CPG-prioritised cases were undertaken by the RM Partners Cancer Hub including the first stage of informed consent indicating the COVID-19 risks. The referring NHS Trusts were requested in advance to provide all the relevant pre-operative clinical information and investigations. At times during the pandemic, national and international guidelines changed for pre-operative isolation and investigation requirements for elective surgery, and the CPG had a dynamic approach to implementing and adjusting guidelines accordingly. All patients were required to have self-isolated for at least 7 days, at certain points, 14 days prior to surgery; have a negative COVID swab 48 h prior to surgery, as well as a non-contrast CT chest confirming no COVID changes when patient likely to require post-operative ICU admission.

### Clinical oversight and governance

The clinical team onsite at the RMH provided clinical oversight of all patients, however those admitted for surgery remained under the direct care of their consultants from the referring NHS Trusts, who ensured their own teams reviewed the patients daily. Clinician indemnity was covered by an NHS England agreement. All major colorectal and anal cancer resections were operated on by at least two consultant surgeons from their referring NHS Trusts donned with full personal protective equipment (PPE) and with adherence to positive flow ventilation protocol as per local guidelines. Open surgery was employed preferentially in the early stages due to concerns about virus aerosolization. Subsequently, an AirSeal® System was used to minimise aerosol contamination during laparoscopic procedures. All patients were anaesthetised by consultant anaesthetists and all resectional surgeries were admitted to critical care post-operatively. All patients in the post-operative period were reviewed daily by a consultant surgeon with the support of senior registrars and an advanced nurse practitioner, in addition to resident medical officers at both surgical site providers, with access to a designated consultant surgeon on-call for out-of-hours emergencies.

Patient demographics, clinicopathological characteristics and post-operative outcomes were recorded in a prospectively maintained database including electronic and medical records, endoscopic, radiological, histopathology reports and discharge summaries. A clinical governance team was established within the RM Partners Cancer Hub with weekly review of audit data and theatre activity including cancellations and post-operative complications. All consultants from involved NHS Trusts were invited to join in the RM Partners Cancer Hub surgical audit monthly online meetings where all surgical cases, complications, 30-day return to theatres and 30-day re-admissions were discussed. All patients received a 28-day post-discharge telephone-based welfare check, from the RM Partners Cancer Hub, where readmission, return to theatre and absence or not of COVID-19 symptoms, was ascertained.

All aspects of inception and implementation of the RM Partners Cancer Hub, including data collection and dissemination was approved by the Institutional Review Board of RMH.

### Data of interest

This study assessed the performance of the established Hub and the impact on theatres utilisation. The outcome of patients treated within the network was collected. Baseline patient characteristics, tumour-related information, and perioperative events were obtained from a prospectively maintained database. Thirty-day complications were graded according to the Clavien–Dindo classification.

### Statistical analysis

Categorical data are reported as absolute values (percentages), whereas continues data are presented as mean ± standard deviation (SD).

## Results

The RM Partners Cancer Hub was established on April 1st 2020. Seven NHS Trusts across north to south west London participated (Fig. 1). This comprised of 7 NHS Trusts which deliver care for a population of over 5 million people.

### Cancer hub performance

176 patients were referred through the online referral form for discussion at the CPG meeting during the peak of the first wave of COVID-19. This period lasted from April 1st to June 30th. The mean time from referral to CPG discussion was 5.7 ± 0.7 days, from referral to anaesthetic assessment was 9.0 ± 1.3 days, and from referral to surgery (all priorities) was 16.3 ± 1.8 days. Of those 176 patients discussed through the CPG meeting, eight patients did not subsequently undergo surgery at RM Partners Cancer Hub during the aforementioned time period. 4 patients were deemed not fit from an anaesthetic point of view; two patients had disease progression at the time of CPG discussion or restaging just prior to surgery, and following MDT discussion, referred back to their referral centre for alternative treatment; one patient opted not to undergo surgery and one patient pre-operative CT chest showed indeterminate features for COVID and therefore surgery was delayed and ultimately underwent surgery in their referral centre after the Cancer Hub dissolution.

### Theatre utilisation

Ultimately 168 patients underwent colorectal or anal cancer surgery at the RM Partners Cancer Hub during the study period; 12 patients were categorised as priority 1b, 142 patients were priority 2 and the remaining 14 were priority 3. 88 patients were operated on in the RMH, and the remainder 80 in the BCH. Thirty-five consultant surgeons participated in the surgeries, with the patient’s primary consultant leading their operation in all cases (Table 1).

**Table 1:** Surgical case volume from London NHS Trusts participating in the RM Partners Cancer Hub

NHS trust	Number	Percentage
Chelsea and Westminster Hospital	45	27%
Royal Marsden Hospital	31	18%
London North West University Healthcare NHS Trust / St Mark’s Hospital	26	15%
Hillingdon Hospital	23	14%
Croydon University hospital	21	12%
Epsom and St Helier Hospital	15	9%
St George’s Hospital	7	4%

Approximately five percent of cases were cancelled within 48 h prior to surgery, and performed at a later stage, meaning that ninety-five percent of the 239 available theatre sessions (each session comprised of a four-hour operating schedule) were utilised. All cancellations were due to patient factors and not operational factors such as lack of critical care beds or staffing issues. 3 patients were cancelled due to concerns of pre-operative CT chest showing indeterminate features suggestive of COVID-19, with negative pre-operative COVID swab. 1 patient expressed wishes of not wanting to go ahead with surgery at the time of admission and 1 other was found to have urosepsis shortly after being admitted. 1 patient was found not to be self-isolating, 1 patient required further investigations, namely cardiac, and in one other case, the surgeon became unexpectedly unavailable to perform the case. In all eight cases, surgery went ahead between 2 and 4 weeks after initial cancellation and within the aforementioned time period.

### Operative outcomes

Patient and tumour characteristics with post-operative outcomes are shown on Table 2. Around three quarters of patients operated upon underwent colonic resection, of which 47% were left-sided resections, 34% right-sided resections and 12% beyond total mesorectal excision (TME) surgeries. Around 79% of all resectional surgeries were performed via laparotomy, while the remainder 21% were performed either robotic or laparoscopically.

**Table 2:** Patient characteristics, time to surgery, operative details and outcomes from surgeries performed by seven participating NHS Trusts at RM Partners Cancer Hub (values given as mean ± SD)

Baseline characteristics (*n* = 168)	
Age > 65	82 (48.8%)
Men	73 (43.5%)
Body mass index (BMI) > 30	105 (62.5%)
Smoker	73 (43.4%)
The American Society of Anaesthesiologists (ASA) Physical Status Classification	
1	13 (7.7%)
2	83 (49.4%)
3	70 (41.7%)
4	2 (1.2%)
Time (days) from Royal Marsden Cancer Hub referral to surgery	
Overall	16.3 ± 1.8
Category 1a	N/a
Category 1b	7.3 ± 1.2
Category 2	17.0 ± 12.3
Category 3	13.6 ± 11.0
Tumour site	
Right colon	37 (22%)
Transverse colon	10 (5.9%)
Left colon	41 (24.4%)
Rectum	53 (31.5%)
Anus	16 (9.5%)
Other	11 (6.5%)
Case mix and type	125 (74.4%)
Resectional	
Right colonic	42
Left colonic/anterior	59
Right and left sided	6
Beyond Total Mesorectal Excision (TME)	15
Small bowel/ovary	3
Non-resectional	
Examination Under Anaesthetic (EUA) with biopsies ±	43 (25.6%)
Colonoscopy or Flexi-Sigmoidoscopy	18
Excision of lesion ± endoscopically	5
Defunctioning stoma	5
Stoma reversal	5
Diagnostic laparoscopy	2
Stoma refashioning	1
Transanal Minimally Invasive Surgery (TAMIS)	6
Excision of abdominal wall mass	1
Surgical approach for resectional surgery (*n* = 125)	
Open	99 (79.2%)
Laparoscopic	22 (17.6%)
Robotic	4 (3.2%)
Surgical outcome for resectional surgery (*n* = 125)	
Anastomosis, without stoma	73 (58.4%)
Anastomosis, with stoma	22 (17.6%)
Stoma with no anastomosis	30 (24%)
Length of stay (days)	
Overall	7.1 ± 1.0
Open	9.3 ± 5.9
Laparoscopic / Robotic	7.0 ± 4.7
30-day post-operative Clavien–Dindo complications	
IIIA	5 (3%)
IIIB	1 (0.6%)
IV	0 (0%)
V	1 (0.6%)

Seven patients (4.2%) developed Clavien–Dindo IIIA and above complications. Of these, four patients required radiological-guided drainage of post-operative collection and one required coronary stenting following post-operative myocardial infarction. One patient developed anastomotic leak 13 days after an open right hemicolectomy and returned to theatre for washout and stoma formation. One patient died from ischaemic bowel following myocardial infarction approximately three days following an open anterior resection (30-day mortality rate of 0.6%). None of the patients developed COVID-19 specific complications post-operatively and up to 28 days post-discharge.

Three patients (1.8%) required re-admission to their local hospital, within 30 days post-discharge. 1 patient suffered from urosepsis, 1 patient from bloodstream septicaemia and 1 other from nausea and vomiting. In all 3 cases, patients were managed with pharmacological treatment.

## Discussion

The SARS-CoV-2 pandemic deeply affected surgical practice globally, with a reduction of emergency admissions and surgical procedures—for several reasons, including staff shortages, healthcare workers fear of infections [3, 7, 14–18]—eventually impacting patients suffering from colorectal cancer. [2, 19, 20]

Evidence supports the feasibility and safety of surgery for common colorectal conditions that, even if non-emergent, might impair patient’s quality of life and long-term survival if treatment is delayed [21–23]. However, it is important that strategies are developed to mitigate the risk of complications related to perioperative SARS-CoV-2 infection [11, 15, 20–22, 24–29]—which is reported to have a detrimental effect on short-term outcomes of surgery [30].

With the arrival of the subsequent waves of COVID-19, and with an unforeseen future of possible lockdowns, the RM Partners Cancer Hub offers an opportunity to model similar cancer or elective surgery “hubs” for streamlined delivery of elective care in COVID-free settings. Risk modelling predicts that a 3-month delay to elective surgery for all cancers could cause almost 5000 deaths per year in England alone and supersedes the risk of COVID-19 even in an age-adjusted population, in terms of life-years lost^9^. Delays to diagnostics and endoscopic or surgical treatments result in a higher burden of advanced and aggressive tumours, as well as in those who present with obstruction or perforation, with downstream negative effects on survival rate and oncologic outcomes [31]. Thus, there is undoubtedly a rationale for ensuring continuity of cancer care at the current and future times.

The RM Partners Cancer Hub was created as an innovative attempt by the colorectal surgery network in west London to deliver cancer care in the midst of the COVID-19 pandemic. The details herein illustrate the steps taken to ensure a robust centralisation process, upholding high standards of clinical governance, whilst retaining individual surgeon autonomy over the care of their own patients, albeit outside of their usual geographical sites of employment. Despite significant logistic and operational challenges, the immediacy of the pandemic allowed quick troubleshooting, ensuring delivery of cancer care in an appropriate, safe and efficient way in one of the biggest worldwide healthcare crisis of this century.

Of note is that for the category 1b, which refers to patients requiring life-saving surgery within 72 h; the median time from RM Cancer Hub referral to surgery was 7.3 ± 1.2 days. This clearly falls outside the NHS recommended time frame. The reason behind it was that some of the category 1b patients were only discussed formally in the CPG after they had their surgery, as CPG meetings were held once weekly, and some cases couldn’t wait until the meeting occurred. In these specific cases, informal discussions between senior members of the CPG Clinical Lead and respective Consultants took place in advance and a joint decision was made to proceed with surgery without further delay.

The logistic challenges that were encountered during the inception of the Cancer Hub were overcome with creative thinking, dynamism and the support of NHS stakeholders, patients and clinicians alike. The majority of patients had open surgery; this reflects the case mix of advanced cancers that are treated within the network, but more accurately reflects the concerns raised about aerosolising procedures at the time. [16, 17, 32–35]. However, the safety of minimally invasive surgery during the pandemic is now accepted [10, 32, 36]. Robotic surgery was almost entirely side-lined to facilitate swifter operations. Digital and virtual means of communication were indispensable in the functioning of the network. Teams and patients quickly got used to new video-conferencing and online referral pathways, and this actually improved levels of participation as well as documentation for audit, whilst allowing the social distancing required to prevent virus transmission. Participation from each trust was important, so that stakeholders, especially clinicians, felt equally accountable and represented at each step of the process. A key and fundamental feature of the model was equity in the theatre allocation process, so that each patient was allocated a surgical slot on a real-time theatre calendar based on need by consensus, and their surgeons were facilitated to travel to the designated surgical sites around their other workplace commitments. The fairness and transparency in this process ensured a positive effect on the whole network’s commitment to the RM Partners Cancer Hub and is reflected in the fact that 35 surgeons performed 168 surgeries over a 13-week time period, without any delays to patient care.

Despite the overwhelming drawbacks of the COVID-19 pandemic, a few positive changes arose. The eagerness to embrace telemedicine is undoubtedly one of the greater achievements in healthcare over the last year and may improve patient input, decrease non-attendances and generate cost savings within the system, as well as national economic savings due to reduced patient absenteeism. Telemedicine also allows those living remotely or with mobility difficulties increased options for accessing care [2, 11, 37]. It must also be highlighted that one of the most important achievements of this collaboration was that no patient developed a nosocomial COVID-19 infection post-operatively, at a point in the pandemic when up to 15% of inpatient COVID-19 infections were hospital-acquired [38]. During the time-period of the hub, there was a small percentage of inpatients, at either of the two hospital sites, COVID-19 positive. This demonstrates that centralising cancer care to elective-only or designated COVID-free sites can reduce COVID-19 risk to patients who are already vulnerable by virtue of their cancer diagnosis.

## Conclusion

The RM Partners Cancer Hub offered elective surgical care for patients with colorectal and anal cancer in a centralised, timely and efficient manner, with acceptable post-operative outcomes and no increased risk of contracting COVID-19 during their inpatient visit. This model may be used in future for when centralising care needs to be implemented in an expeditious fashion.
